# The impact of the COVID-19 pandemic on elective laparoscopic cholecystectomy: A retrospective Cohort study

**DOI:** 10.3389/fsurg.2022.990533

**Published:** 2022-12-07

**Authors:** George Demetriou, Kasun Wanigasooriya, Ahmed Elmaradny, Ammar Al-Najjar, Mohammad Rauf, Alicia Martin-Jones, Mohamed Saad Aboul-Enein, Steven J Robinson, Anthony Perry, Martin S Wadley, Moustafa Mourad

**Affiliations:** ^1^ Department of Upper Gastrointestinal and Bariatric Surgery, Worcestershire Acute Hospitals NHS Trust, Charles Hastings Way, Worcester, United Kingdom; ^2^ Department of General Surgery, Queen Elizabeth Hospital, University Hospitals Birmingham NHS Foundation Trust, Edgbaston, Birmingham, United Kingdom; ^3^ College of Medical and Dental Science, University of Birmingham, Vincent Drive, Edgbaston, Birmingham, United Kingdom; ^4^ General Surgery Department, Faculty of Medicine, Tanta University, Tanta, Egypt

**Keywords:** COVID-19, laparoscopic cholecystectomy, clavien-Dindo, retrospective study, United Kingdom

## Abstract

The coronavirus disease 2019 (COVID-19) pandemic had a significant impact on elective surgery for benign disease. We examined the effects of COVID-19 related delays on the outcomes of patients undergoing elective laparoscopic cholecystectomy (LC) in an upper gastrointestinal surgery unit in the UK. We have analysed data retrospectively of patients undergoing elective LC between 01/03/2019 to 01/05/2019 and 01/04/2021 to 11/06/2021. Demographics, waiting time to surgery, intra-operative details and outcome data were compared between the two cohorts. Indications for surgery were grouped as inflammatory (acute cholecystitis, gallstone pancreatitis, CBD stone with cholangitis) or non-inflammatory (biliary colic, gallbladder polyps, CBD stone without cholangitis). A *p* value of <0.05 was used for statistical significance. Out of the 159 patients included, 106 were operated pre-pandemic and 53 during the pandemic recovery phase. Both groups had similar age, gender, ASA-grades and BMI. In the pre-pandemic group, 68 (64.2%) were operated for a non-inflammatory pathology compared to 19 (35.8%) from the recovery phase cohort (*p *< 0.001). The waiting time to surgery was significantly higher amongst patients operated during the recovery phase (*p *= 0000.1). Less patients had complete cholecystectomy during the pandemic recovery phase (*p *= 0.04). There were no differences in intraoperative times and patient outcomes. These results demonstrate the impact of COVID-19 related delays to our cohort, however due to the retrospective nature of this study, the current results need to be backed up by higher evidence in order for strong recommendations to be made.

## Introduction

The coronavirus disease 2019 (COVID-19) pandemic led to the cancellation of millions of planned operations worldwide (1). Patients suffering from benign conditions such as gallstone disease, inflammatory bowel disease or osteoarthritis saw their operations postponed due to perceived risks from peri-operative COVID-19 infection and due to reallocation of resources to care for COVID-19 patients. In the United Kingdom, by March 2021 around 5 million patients were waiting for surgery, with more than 436000 waiting for more than a year (2). As healthcare systems recover from the acute phase of the COVID-19 pandemic, the collateral impact of this pandemic due to cancelled surgery on patients with benign conditions becomes apparent (2, 3).

Delay for surgery is likely a strong contributing factor for more complicated gallstone related presentations like recurrent attacks of cholecystitis, choledocholithiasis and pancreatitis and can expose the patients to risks from procedures like IR drainage and Endoscopic Retrograde Cholangiopancreatography (ERCP) and to higher risks during surgery (4). Before the pandemic, the recommendation by the Association of Upper Gastrointestinal Surgeons (was to perform laparoscopic cholecystectomy (LC) during the index admission or within one week of an attack of acute cholecystitis (AC) and for those with mild to moderate gallstone pancreatitis (AGP) within the index admission or within 2 weeks of presentation (5). During the pandemic the guidance changed to conservative management-only in both AC and AGP, and cholecystostomy was recommended for the septic patients with gallbladder empyema (6). Consequently, when most centres resumed elective surgery, many patients had been waiting for more than a year to undergo cholecystectomy (2, 3). Many surgeons perceived that these cases will be associated with longer operative times, increased intraoperative difficulty and prolonged post-operative recovery times. Such challenging cases could also slow-down the post-pandemic recovery plans of surgical departments faced with long waiting lists of patients awaiting LC and have an impact on other more urgent waiting lists such as those for cancer surgery.

We set out to evaluate a single-centre's experience of elective cholecystectomy following the acute phase of the pandemic.

## Methods

### Study design and setting

A retrospective data analysis of patients undergoing elective LC at a district general hospital in the UK was undertaken. The study was registered with the local clinical governance team (ID: 11027). Research ethics committee approval was not required for this retrospective observational study and this was confirmed using the UK, Health Research Authority “Is my study research?” online decision tool (http://www.hra-decisiontools.org.uk/research). Two distinct cohorts of patients were identified using local theatre patient management software (“Bluespier Theatres” System). The first cohort comprised of patients undergoing elective LC before the pandemic between 01/03/2019 to 01/05/2019 [Pre-pandemic cohort (PP-2019)]. The second cohort comprised of those patients operated between 01/04/2021 and 11/06/2021 [Pandemic recovery phase cohort (PRP-2021)]. These patients were amongst the first to be operated on after elective surgery for benign conditions resumed at our organisation following the acute phase of the pandemic in April 2021. Patients undergoing emergency cholecystectomy were not included in this study. All operations were performed by a consultant upper gastrointestinal surgeon or by a trainee surgeon under direct consultant supervision.

## Data collection

Data were collected by five clinicians from the study team and each entry was independently verified by at least two members. Data were obtained retrospectively using electronic patient records. Demographic data collected included age, gender, American Society of Anaesthesiologists (ASA) grade and body mass index (BMI). Data were also collected on indication for surgery and grouped by pathology; non-inflammatory (biliary colic, gallbladder polyps and CBD stone without associated cholangitis) or inflammatory (cholecystitis, pancreatitis and CBD stone with cholangitis). The date each patient was added to the waiting list and the date of surgery were used to estimate the waiting time for surgery in weeks. Intra-operative details such as operative time (calculated in minutes from skin incision to skin closure), intra-operative findings, requiring open conversion and use of drain were obtained using digitally archived operation notes and a local theatre patient management software. The procedure was defined as a complete cholecystectomy if the whole gallbladder was successfully removed, subtotal cholecystectomy where part of the Gallbladder was left behind or failed surgery where the procedure was abandoned due to intraoperative difficulty. Data were also collected on length of stay (LoS), postoperative complications (Clavien-Dindo classification; Supplementary Table S1), return to theatre (RTT) and 30-day readmission.

### Data analysis

The data were collated using Excel (Microsoft Corporation, USA). Categorical data were presented as integers and percentages. Non-parametric data were summarised using median and interquartile range (IQR). Univariate analysis was performed using Chi-square or Fisher's exact test. Hypothesis testing was performed using Mann-Whitney U-test. Statistical analysis was performed using SPSS V26 (IBM, USA). A *p-*value of <0.05 was assigned as the level of statistical significance.

## Results

### Combined results

In total, 159 patients who underwent elective LC were included in the analysis. Approximately two thirds (*n* = 106; 66.7%) were operated before the pandemic (PP-2019 group) and a third (*n* = 53; 33.3%) were operated during the pandemic recovery phase (PRP-2021 group; Table 1). The median age was 56 (IQR: 43–68) years of age. The majority were female (*n* = 115; 72.3%). Most patients were ASA 1 (*n* = 21; 13.2%) or 2 (*n* = 105; 66%).

**Table 1:** Patient characteristics.

	Pre-pandemic (2019)		Recovery phase (2021)			
	N	%	N	(%)	Total	*p*-value^a^
Total	106	(100%)	53	(100%)	159	-
Age						
Median	56	years	57	years		
IQR	21–68	years	18–69	years		
<60	67	(63.2%)	31	(58.5%)	98	0.564
>=60	39	(36.8%)	22	(41.5%)	61	
Gender						
Male	29	(27.4%)	15	(28.3%)	44	0.9
Female	77	(72.6%)	38	(71.7%)	115	
ASA grade						
1	13	(12.3%)	8	(15.1%)	21	0.213^b^
2	73	(68.9%)	32	(60.4%)	105	
3	14	(13.2%)	13	(24.5%)	27	
4	0	(0%)	0	(0%)	0	
Not known	6	(5.7%)	0	(0%)	6	
<25	17	(16%)	13	(24.5%)	30	0.131^c^
25.0–29.9	37	(34.9%)	11	(20.8%)	48	
≥30	50	(47.2%)	29	(54.7%)	79	
Not known	2	(1.9%)	0	(0%)	2	
**Non-inflammatory**	**68**	**(** **64.2%)**	**19**	**(** **35.8%)**	**87**	<0.001^d^
Biliary colic	57	(53.8%)	16	(30.2%)	73	
Gallbladder polyps	11	(10.4%)	3	(5.7%)	14	
Obstructive jaundice	5	(4.7%)	2		7	
**Inflammatory**	**38**	**(** **35.8%)**	**34**	**(** **64.2%)**	**72**	** **
Acute cholecystitis	20	(18.9%)	19	(35.8%)	39	
Gallstone pancreatitis	13	(12.3%)	10	(18.9%)	23	
Cholangitis	0	(0%)	2	(5.8%)	3	

^a^ Chi-Square test was performed.

^b^ ASA1,2,3 analysed.

^c^ Missing data excluded.

^d^ Non-inflammatory vs. inflammatory.

### Similarities and differences between the two groups

#### Pre-operative

There was no difference between age, gender, ASA grade and BMI on univariate analysis between the two cohorts. However, there was a significantly higher proportion of inflammatory pathology noted in the patients undergoing cholecystectomy following the acute phase of the pandemic (64.2% vs. 38.2%; *p *< 0.001). The proportion of patients undergoing elective surgery for cholecystitis was approximately twice that of before the pandemic (35.8% vs. 18.9%).

The waiting time to surgery was significantly higher in the pandemic recovery cohort compared to the pre-pandemic one 20 (8–28) vs. 8 (8–15) weeks (U = 1741; *p *= 0.0001).

#### Intra-operative

A Mann-Whitney U test revealed that there was no significant difference (U = 2847; *p *= 0.890) in the intraoperative times between the two cohorts. Surgeons successfully performed complete cholecystectomy in a higher proportion of patients before the pandemic (99.1% vs. 92.5%) (*p *= 0.04). In the pandemic recovery cohort more procedures were abandoned and more patients underwent a subtotal cholecystectomy (7.1%). No procedures were abandoned in the pre-pandemic cohort and one subtotal was performed (0.9%). The open conversion rate was 1.9% (*n* = 1) in the pandemic recovery phase and this was due to bleeding. None were converted to open in the first cohort. Whilst a higher proportion of intra-operative drain insertion was noted following the pandemic (11.3% vs. 8.5%), univariate analysis revealed this not to be a statistically significant difference (*p *= 0.573) (Table 2).

**Table 2:** Intraoperative details.

	Pre-pandemic (2019)		Recovery phase (2021)			
	N	%	N	(%)	Total	*p*-value
Total	106	(100%)	53	(100%)	159	–
Operative time						
Median	64	minutes	65	minutes	–	–
IQR	25–80	minutes	25–90	minutes	–	–
<60 min	47	(44.3%)	26	(49.1%)	73	0.574^a^
>=60 min	59	(55.7%)	27	(50.9%)	86	
Surgery performed						
Complete cholecystectomy	105	(99.1%)	49	(92.5%)	154	0.04^c^
Subtotal or abandoned	1	(0.1%)	4	(7.5%)	5	
Open conversion						
Yes	0	(0%)	1	(1.9%)	1	–
No	106	(100%)	52	(98.1%)	158	
Drain (s)						
Yes	9	(8.5%)	6	(11.3%)	15	0.573^b^
No	97	(91.5%)	47	(88.7%)	144	

^a^ Chi-square test was performed.

^b^ Fisher's exact test was performed.

^c^ Analysis between complete cholecystectomy vs. subtotal or abandoned.

.

#### Post-operative

Approximately two thirds (*n* = 68; 64.2%) of patients underwent day case surgery in the pre-pandemic group compared to half (*n* = 27; 50.9%) in the pandemic recovery group. Whilst univariate analysis demonstrated this not to be a statistically significant difference (*p *= 0.109) the maximum LoS for the latter group was 13 compared to 5 of the former. There was no significant difference in overall complication rates between the two groups (8.5% vs. 7.6%; *p *> 0.05). However, the proportion of patients requiring return to theatre was slightly higher in the pandemic recovery cohort (3.8% vs. 1.9%). The 30-day readmission rate was lower before the pandemic (7.5% vs. 11.3%). One patient from each cohort attended the Surgical Assessment Clinic with postoperative pain but did not require admission (Table 3).

**Table 3:** Post-operative complications.

	Pre-pandemic (2019)		Recovery phase (2021)			
	N	%	N	(%)	Total	*p*-value
Total	106	(100%)	53	(100%)	159	-
Day case						
Yes	68	(64.2%)	27	(50.9%)	95	0.109^a^
No	38	(35.8%)	26	(49.1%)		
Complications (Clavien-Dindo Classification)						
**Grade I**	**1**	**(0.9%)**	**0**	(0%)	**1**	^c^1.000^b^
Deranged LFTs	1	(0.9%)	0	(0%)	1	
**Grade II**	**5**	**(4.7%)**	**2**	**(3.8%)**	**7**	** **
Wound infection	2	(1.9%)	1	(1.9%)	3	
Collection	3	(2.8%)	1	(1.9%)	4	
**Grade IIIa**	**1**	**(0.9%)**	**0**	**(0%)**	**1**	** **
Retained stone	1	(0.9%)	0	(0%)	1	
**Grade IIIb**	**2**	**(1.9%)**	**2**	**(3.8%)**	**4**	** **
Bleeding	0	(0%)	1	(1.9%)	1	
Bile duct injury	0	(0%)	1	(1.9%)	1	
Bile leak	1	(0.9%)	0	(0%)	1	
Bowel injury	1	(0.9%)	0	(0%)	1	
**Grade IV or V**	0	(0%)	0	(0%)	0	
**None**	**97**	**(91.5%)**	**49**	**(92.4%)**	**146**	** **
Return to theatre (Grade IIIb)						
Yes	2	(1.9%)	2	(3.8%)	4	1.000^b^
No	104	(98.1%)	51	(96.1%)	155	
30-day readmission						
Yes	8	(7.5%)	6	(11.3%)	14	1.000^b^
No	98	(92.5%)	47	(88.7%)	145	

^a^ Chi-square test was performed.

^b^ Fisher's exact test was performed.

^c^ Analysis between all complications vs. none. LFT, liver function tests.

.

## Discussion

**Figure 1:**
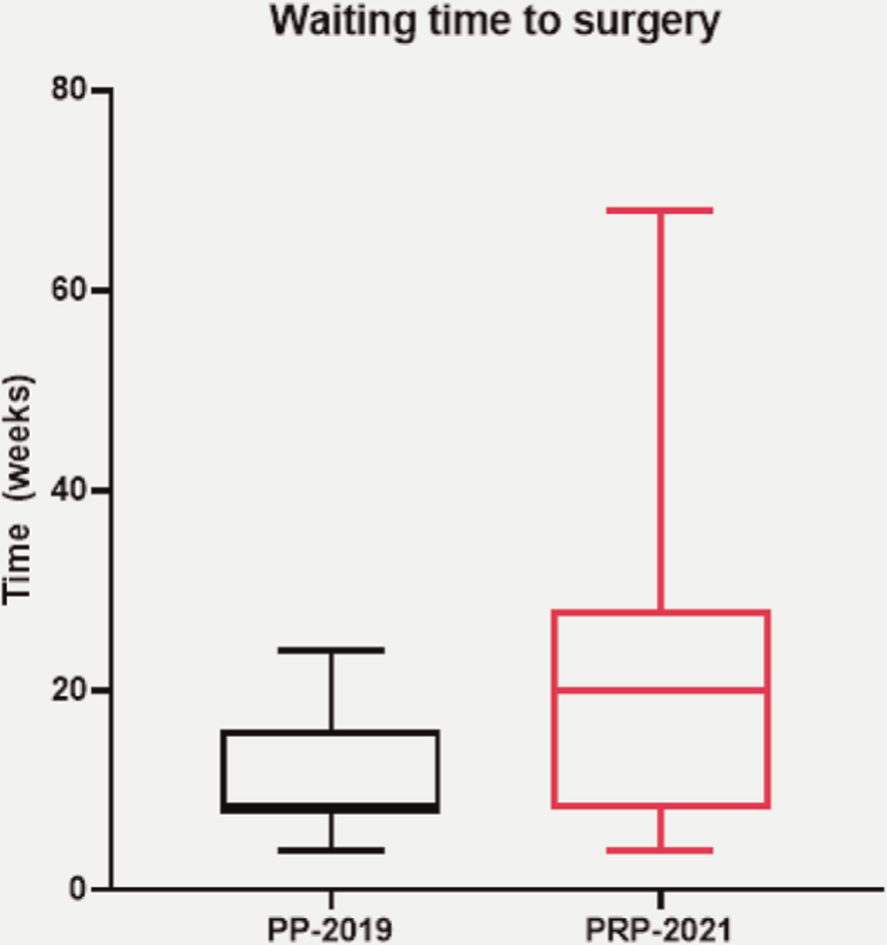
Waiting time to surgery between the two groups. PP-2019 - Pre-pandemic group; PRP-2021: Pandemic recovery phase group.

This retrospective cohort study evaluated the characteristics and outcomes of patients undergoing elective LC for benign gallbladder disease before the Covid-19 pandemic and during the pandemic recover phase, in a single secondary care hospital in the UK. Overall, patient demographics in our study were comparable to those reported in other literature (3, 6, 7). However, the proportion of patients who were ASA-3 in our sample was higher at 17% compared to 7.9% reported by previous studies (7), the majority (54.7%) of our patients were operated for biliary colic. However, the proportions were different between the two groups. Before the pandemic (PP-2019 group), 68 (64.2%) of patients were operated for biliary colic compared to only 19 (35.8%) in the pandemic recovery cohort (PPR-2021) (*p* < 0.001). Previous studies conducted before the pandemic have also reported that the most common indication for elective laparoscopic cholecystectomy is biliary colic (4). The low rates of biliary colic observed during the pandemic recovery phase could be attributed to long waiting times and patients developing more complications from their gallstone disease. In fact, our data confirmed that the waiting time to surgery were significantly higher for patients in the 2021 cohort compared to the 2019 one (Figure 1). The waiting times were calculated from the time the patient was added on the waiting list for surgery which might underestimate the true duration of symptoms. However our data could not capture the waiting time to surgery from the onset of symptoms accurately as many patients were already seen in other hospitals in the region, GP practice or private sector.

A significantly lower proportion of patients underwent a complete cholecystectomy in the pandemic recovery phase (*n* = 49; 92.5%) compared to the those operated before the pandemic (*n* = 106; 99.1%; *p *= 0.04). From those five (3.1%) who haven't had a complete cholecystectomy, three underwent a subtotal cholecystectomy and two had their operation abandoned. The overall rate of subtotal cholecystectomy was 1.8%. This is lower than previously reported rates of 4.5%–16.2% in the literature (8, 9). The subtotal cholecystectomy rate in the recovery phase (4.1%) was higher compared to before the pandemic (0.9%). Whilst no procedures were abandoned in patients operated before the pandemic two were abandoned in patients operated during the pandemic recovery phase. In both patients, concerns of a choledocoduodenal fistula led to surgery being abandoned; one was referred to a tertiary hepatobiliary unit and the other for further imaging.

The overall rate of conversion to open surgery was 0.6% and was lower compared to previously published rates (1.6%–8.9%) (3, 7). Furthermore, there was no significant difference between the rates of open conversion rates or median operating times between the two groups. The threshold for drain placement varied between individual surgeons. Whilst the latter might not necessarily reflect an adverse event, drain insertions during LC can lead to increased length of stay and is associated with higher rates of wound infection (10). More patients received a drain at surgery following the pandemic (11.3% vs. 8.5%, *p *= 0.573). One study reported a drain insertion rate of 30.8% during laparoscopic cholecystectomy for patients with cholecystitis during the peak of the Covid-19 pandemic as 30.8% (3). Our findings may be attributable to the higher incidence of acute cholecystitis (35.8% vs. 18.9%) and more inflammatory intraoperative findings (thickened gallbladder, abscesses, empyema, adhesions) in the PRP-2021 group. However, it may also reflect the higher degree of caution and wariness amongst surgeons operating in patients within pandemic recovery phase group.

The rate of day case surgery in this sample was 60%. Whilst this was higher before the pandemic it was not statistically significant (64.2% vs. 50.9%; *p* > 0.05). The British Association of Day Surgery standards recommends at least 60% of all elective LC be done as day cases (11). Whilst this target was met for patients in before the pandemic it was however, not met for patients operated during the pandemic recovery phase. However, the proportion of patients undergoing day case surgery in either group was lower than previously reported in the literature (7, 12). Several studies identified predictors for failed discharge in day case surgery for LC. A “Cholecystectomy as a Day Case score” was developed in a study to predict the likelihood of successful day-case cholecystectomy. Factors associated with failed day-case cholecystectomy included older age, male sex, complicated choledocholithiasis, higher ASA Scores and previous admission with gallstone disease (13). Several patients during the pandemic recovery phase had one or more of these factors which could have contributed to the lower day-case surgery rate in this group.

There was no significant difference between postoperative complication rates between the two groups. However, there was a trend for worse outcomes during the pandemic recovery phase. For example, the proportion of patients that required return to theatre (Grade IIIb complication) was higher 3.8% compared to 1.9% before the pandemic. Due to the small number of patients in each cohort the complication rate might appear to be higher than the actual number. The 30-day re-admission rates overall in this study was 8.8% and this was compliant with UK standards (<10%) (5). It was higher in the recovery phase group at 11.3% compared to 7.5% in the pre-pandemic group. Previously published reports from the UK and USA reported the 30-day readmission rates between 5.4%–7% (8, 14, 15) and the most commonest complain was non-specific abdominal pain. This was the same reason for most readmissions in our cohort too.

The main limitation of this retrospective study was the relatively small sample size. Other limitations included the lack of inclusion of emergency cholecystectomies. However, our aim was to report the impact of COVID-19, on elective services during the recovery phase. Due to the retrospective nature of this study, we were unable to grade the intraoperative difficulty using an objective tool such as the Nassar operative difficulty scale (4). Greater intraoperative difficulty has been associated with worse outcomes following LC (10) and may therefore, be a confounder for observed outcomes across the two groups. Despite these limitations our study was one of the first to report the impact on the Covid-19 pandemic on elective laparoscopic cholecystectomy and variations in patient pathology, surgical practices and similarities between outcomes between pre-pandemic and recovery phase patients.

As the UK NHS emerges from the pandemic, there is increased pressure to maintain the high-quality standards of clinical practice and to reduce the waiting times for definitive treatment across all specialties. However, additional resources must be allocated to ensure more theatre capacity, staffing and elective beds including high dependency care are available to cater to more complex cases arising because of delayed surgery. Patient numbers allocated to elective lists may also need adjusting to compensate for the higher complexity of surgery. In addition, regular auditing is vital to ensure the practice meets the local pre-pandemic standards and national standards. The immediate and long-term impact on patients with benign conditions such as gallstone disease who had their definitive treatments delayed due to the pandemic warrants further attention and research.

## Data Availability

The original contributions presented in the study are included in the article/**Supplementary Material**, further inquiries can be directed to the corresponding author/s.
